# Computational materials design of crystalline solids†
†Electronic supplementary information (ESI) available: An extended reading list taken from a snapshot of the Mendeley Group on Materials Design available at https://www.mendeley.com/groups/8113991/materials-design. See DOI: 10.1039/c5cs00841g



**DOI:** 10.1039/c5cs00841g

**Published:** 2016-03-18

**Authors:** Keith T. Butler, Jarvist M. Frost, Jonathan M. Skelton, Katrine L. Svane, Aron Walsh

**Affiliations:** a Centre for Sustainable Chemical Technologies and Department of Chemistry , University of Bath , UK . Email: a.walsh@bath.ac.uk; b Global E3 Institute and Department of Materials Science and Engineering , Yonsei University , Seoul , Korea

## Abstract

The modelling of materials properties and processes from first principles is becoming sufficiently accurate as to facilitate the design and testing of new systems *in silico*. Computational materials science is both valuable and increasingly necessary for developing novel functional materials and composites that meet the requirements of next-generation technology. A range of simulation techniques are being developed and applied to problems related to materials for energy generation, storage and conversion including solar cells, nuclear reactors, batteries, fuel cells, and catalytic systems. Such techniques may combine crystal-structure prediction (global optimisation), data mining (materials informatics) and high-throughput screening with elements of machine learning. We explore the development process associated with computational materials design, from setting the requirements and descriptors to the development and testing of new materials. As a case study, we critically review progress in the fields of thermoelectrics and photovoltaics, including the simulation of lattice thermal conductivity and the search for Pb-free hybrid halide perovskites. Finally, a number of universal chemical-design principles are advanced.

## Abstract 2

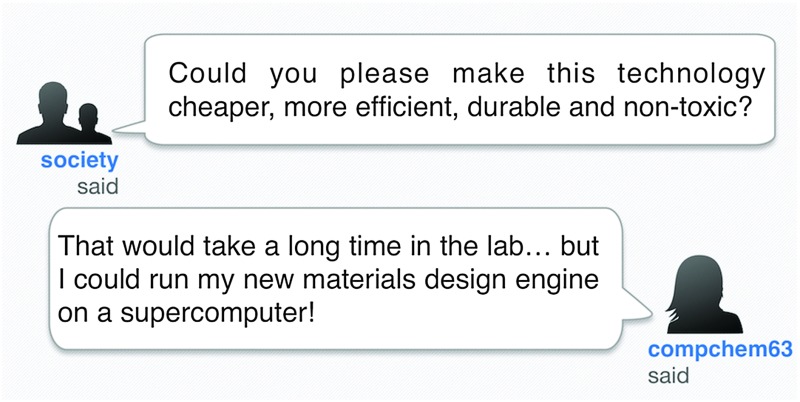
Recent advances in the computational techniques and procedures for the design of functional materials are reviewed.

Key learning points

(1) First-principles atomistic materials modelling is versatile and can be quantitative and predictive.

(2) A range of tools exist for the simulation and screening of new materials even before they have been made in the laboratory.

(3) A clear metric, based on a calculable set of descriptors, is required to facilitate materials discovery and optimisation for specific applications.

(4) Materials modelling can be used to reduce the chemical combinatorial space and to identify promising candidate structures and compositions as part of a holistic materials-design procedure.

## 1. Introduction

The rich diversity of naturally-occurring materials has provided a solid foundation for modern science and technology. Our understanding of these materials, and indeed the very concept of chemical bonding, has developed from centuries of research into their chemical and physical responses. We now know that the multifarious properties of materials – including colour, conductivity, magnetism, and reactivity – are intimately related to the chemical composition and crystal structure.^1^ For example, each rocksalt-structured metal oxide is a distinct chemical system with variation in physical properties,^2^ while each polymorph of TiO_2_ has unique properties, owing to differences in the local coordination environments of the cation and anion.^3^

The limitations of known materials are apparent in many technological areas, which are driven by multiple factors including cost, performance and sustainability. Fortunately, the Periodic Table offers immense potential for developing new materials. The number of known materials represents just a small fraction of the possible combinatorial space.^4^ This includes thermodynamically-stable configurations that are not known to occur in nature, as well as metastable configurations that have become accessible through advances in synthetic materials chemistry.^5^ Metastable materials and composites can have kinetic lifetimes sufficient for practical applications.^6^

The rapid technological increase in computer processor speed and the strategic investment in contemporary supercomputers have supported a renaissance in the fields of computational chemistry and computational materials science. Long-standing approximations can be removed and the constraints of length- and timescales overcome, so that more quantitative and realistic simulations are accessible. One recent example of a technical advancement is the application of full configuration-interaction quantum Monte Carlo simulations to solids, a ‘gold standard’ electronic-structure approach that was previously deemed prohibitively expensive.^7^ Computational materials science has historically been responsive to experiment, whereas now an increasing amount of trust is being placed on materials modelling to guide experiment and provide solutions to real technological challenges.^8^

**Fig. 1:**
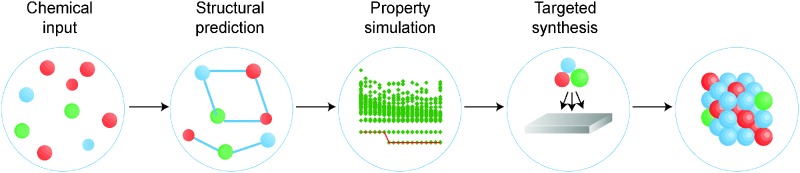
A modular materials-design procedure, where an initial selection of chemical elements is subject to a series of optimisation and screening steps. Each step may involve prediction of the crystal structure, assessment of the chemical stability or properties of the candidate materials, followed by experimental synthesis and characterisation. A material may be targeted based on any combination of properties, for example a large Seebeck coefficient and low lattice thermal conductivity for application to heat-to-electricity conversion in a thermoelectric device. [Reproduced with permission from ref. 4].

There is a strong demand for novel materials with tailored properties – the challenge is to identify them. There has been impressive progress in combinational materials fabrication and characterisation procedures; however, such efforts are usually limited to a two- or three-dimensional parameter space, and may suffer from issues with materials quality, contamination and isolation. The integration of materials simulation into this design procedure (see Fig. 1) can be used both to screen the most promising candidate materials and to expedite the materials characterisation, *e.g.* by providing the spectral signatures required to identify the proposed phases.^9^

In this Tutorial Review, we critically discuss the latest developments in the computational materials design of crystalline solids and its application to the development of thermoelectric and photovoltaic devices based on earth-abundant elements, and attempt to extract a set of universal chemical principles that define a holistic design procedure.

## 2. Computational techniques

Our focus for the computational framework of materials modelling is not the underlying total-energy or property calculator (*e.g.* pairwise interatomic potentials, density-functional theory, or many-body perturbation theory), but the auxiliary techniques and methodology that facilitate the development and optimisation of new materials.

In addition to the electronic structure (electron density and electronic energy levels), most quantum-mechanical treatments of solids also provide a reliable description of the total internal energy of the system. By calculating the lattice vibrations (phonons) around the equilibrium lattice positions, the full range of thermodynamic potentials, including the Gibbs free energy, can be considered.^10^ Such a statistical-mechanical treatment is particularly beneficial when considering the stability of multi-component systems and their possible disproportionation reactions.^11^ It is important to remember that any thermodynamic analysis refers to equilibrium conditions, while a variety of modern synthetic techniques provide access to non-equilibrium stoichiometries and structures, which, despite being metastable, may have a long lifetime under conditions of practical interest.^5,6^

**Fig. 2:**
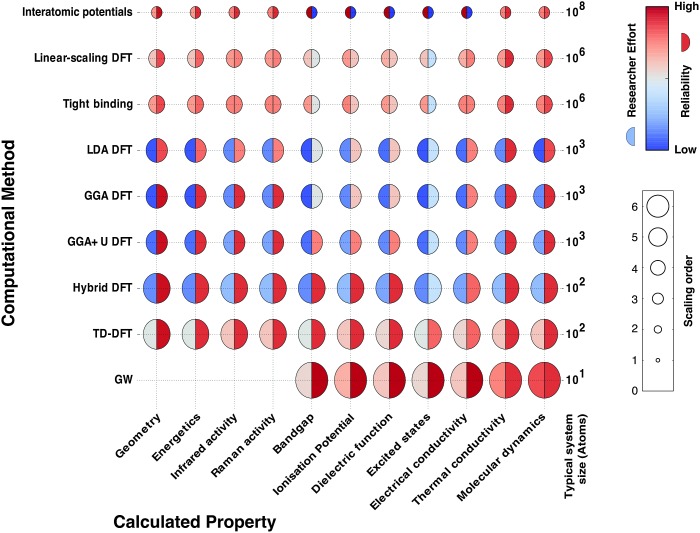
Map showing the accessibility of different calculable material properties for a set of common computational methods. The methods include several flavours of density functional theory (DFT) which differ in the treatment of the quantum mechanical electron–electron interactions (*e.g.* local density approximation (LDA) and generalised gradient approximation (GGA)) as well as empirical tight-binding and many-body *GW* approaches. The circle size corresponds to the scaling of the computational effort with system size, the shading of the left semicircle represents the researcher effort required to use the method, and the shading of the right semicircle represents the reliability of the results from the method. Some properties are currently not calculable with *GW* theory for solids, and thus these circles are omitted.

In Fig. 2 we schematically represent a set of principal techniques and calculable material properties. We have attempted to provide an outsiders' guide to the relative computational expense (size of circle), “difficulty” in terms of researcher effort (left semicircle colour) and reliability (right semicircle colour) of standard methods for predicting properties. There is a well-known trade-off between the accuracy of methods and their computational cost. However, there is much subtlety within this relationship. One aspect less talked about, and much harder to quantify, is the opportunity cost of researcher time. Empirical techniques, though computationally efficient, require material-specific fitting of parameters, a laborious and expert undertaking. Sophisticated electronic structure techniques (*e.g.* linear scaling density functional theory or *GW* theory) require specialist codes and knowledge much closer to the research frontier; the calculations are therefore considerably more hands-on, requiring more researcher expertise and effort. For these reasons, the vast majority of current research uses density functional theory (DFT), the computationally most efficient *ab initio* technique for solids. A virtuous feedback has existed between successful scientific studies, code development and proliferation of research expertise. Our expectation is that the future will see more sophisticated electronic structure methods become integrated into the standard codes, as computational power expands further and algorithms are developed to automate human expertise.

All of the computational methods discussed here are atomistic. The fundamental input to a solid-state calculation which provides an observable quantity is the crystallographic unit cell and the three-dimensional locations of the atoms. For a known material this can come directly from a solved crystal structure, whereas in order to design new materials we must first consider the different ways in which we can generate candidate structures.

### a. Data mining of known structures

Data mining is the analysis of large data sets to identify trends and patterns, which can be used to form predictive empirical rules.

Within materials science, the Materials Project is a commendable effort^12^ built on the open-source *pymatgen* codes. By constructing a systematic database of calculated properties of all known inorganic materials with crystal structures reported in crystallographic databases, it allows for: (i) property screening of known compounds; (ii) simulation of complete phase diagrams; (iii) identification of errors in reported crystal structures; (iv) a comprehensive assessment of the errors associated with different levels of theory. One recent application of data mining has been the search for non-conventional p-type transparent conducting oxides, which highlighted the importance of including cations with valence orbitals that can hybridise effectively with O 2p, including the d^10^ and s^2^ electronic configurations.^13^

A major challenge is how to interpret trends and correlations in the data. Much effort is being put into materials informatics, *e.g.* principal component analysis^14^ and structure–property cartograms.^15^ These techniques attempt to interrogate data and establish relationships from an unbiased viewpoint in an automated manner. However, given the inherent complexity of chemical bonding – even for a single stoichiometry, properties can vary over a large range with just small changes in crystal structure – it is difficult to gauge the general effectiveness of this approach. Most structure–property relationships discussed in the literature tend to consist of post-rationalisation driven by chemical intuition. Some notable examples have included linking superconductivity to structural instabilities (soft phonon modes) and magnetism to two- and three-body connectivity in the underlying atomic networks.^16^

### b. Crystal structure prediction from chemical composition

The standard paradigm of materials modelling is INPUT: structure → OUTPUT: properties. This workflow usually starts with a solved crystal structure from diffraction experiments, providing a well-defined unit cell and a set of atomic positions, which is then locally refined for a given level of theory. The prediction of ground-state crystal structures from chemical composition is much more difficult, as it requires global rather than local optimisation.^17^ Each crystallographic unit cell has six degrees of freedom (the lengths and angles of the three lattice vectors) as well as the three degrees of freedom associated with each of the internal atomic positions. Even with just a few atoms, the number of possible configurations exceeds that which can be systematically investigated. Involvement of materials modelling during the structure solution is an increasingly fruitful area of collaboration.^18^

Global structure optimisation of solids usually relies on stochastic approaches to efficiently sample the relevant areas of configurational space and to provide an approximate but still relevant solution. A wide variety of genetic and evolutionary algorithms, Monte-Carlo sampling techniques, particle-swarm methods and minima-hopping approaches are used.^19^ A substantial amount of human effort has been invested in optimising these algorithms, and the underlying move classes defining possible structural modifications, for reliable and robust identification of thermodynamically-accessible crystalline phases. To avoid algorithmic biases, a fully random structure search can be attempted,^20^ which continuously arranges atoms or moieties randomly in a unit cell without an underlying global optimisation algorithm. While simple in concept, such an approach is prone to failure for complex systems with multi-dimensional potential-energy surfaces.

Several implementations of global optimisation algorithms for solids exist, for example, in the codes XtalOpt,^21^ USPEX,^22^ CALYPSO^23^ and KLMC.^24^ There have been notable successes in the prediction of phase behaviour under extreme conditions, *e.g.* the emergence of new phases of boron and NaCl under high pressure.^22,25^

### c. Chemical analogy

A computationally efficient materials-discovery procedure is to take a given crystal structure and screen all chemically plausible elemental combinations within this lattice. Thereby, previously overlooked or ‘missing’ compounds can be identified. One example is the screening of oxide, oxyhalide and oxysulfide perovskite-structured materials for photoelectrochemical water splitting.^26^ Follow-on work highlighted the effectiveness of genetic algorithms in identifying the best candidates for synthesis from a pool of 19 000 materials.^27^

The causal relationship assumed in such studies is that elements and structure lead to properties, based on which empirical rules are formed. The key to the success of this procedure is the application of relevant and informative descriptors, both for the constituent elements and for the desired material properties. Some useful descriptors for elements, crystal structures and properties are listed in Table 1.

**Table 1:** A list of commonly-used descriptors in materials screening and design

Atom/ion	Structure	Property
Atomic number	Stoichiometry	Seebeck coefficient
Radius	Density	Band gap
Electronegativity	Space group	Ionisation potential
Oxidation state	Lattice parameter	Polarisation
Solid-state energy	Coordination	Magnetic moment
Magnetic moment	Connectivity	Dielectric constant
Polarisability	Bond length	Carrier effective mass

The relationship between composition, structure and properties has been one of the great challenges in solid-state chemistry for almost 100 years.^1^ One successful strategy based on structural analogy is to start from a parent crystal structure and perform site mutations whilst enforcing a consistent electron count per unit cell, notably the 8- and 18-electron rules. As early as 1964, Pamplin^28^ outlined a procedure to derive the plausible compositions of multi-component tetrahedral semiconductors, as will be discussed below in the context of materials for solar cells. A similar strategy was applied to the ABX class of materials that conform to the 18-electrons per unit cell rule.^9^ The authors applied the principles of electron counting to identify 400 unreported, but plausible, compounds. These compounds were then assessed for thermodynamic stability using density functional theory (DFT), yielding 54 stable combinations from which 15 previously unreported materials were then grown and characterised.

An alternative approach is to go directly from chemical composition to physical properties. One useful elemental descriptor is the solid-state energy (SSE),^29^ which has been derived from the ionisation potentials and electron affinities of a series of binary compounds. The concept is that the valence-band maximum of a binary compound is determined primarily by the electronic energy levels of the anion, whilst the conduction-band minimum is determined by the energy levels of the cation. By statistical analysis of a training set of ionisation potentials (IPs) and electron affinities (EAs), the energy levels of common anions and cations were determined. These values can subsequently be applied to new combinations of the constituent elements to estimate band energies and gaps, and hence to assess the potential suitability of the compounds for a range of applications. A similar approach, based on the Mulliken electronegativity of the elements, has been applied to estimate the flat band potentials of metal-oxide materials in the context of photoelectrochemistry.^30^

### d. Inverse design

In many ways, the approaches discussed above are “brute force”: a materials dataset is built and then screened to assess suitability for a desired application. A more elegant procedure is where a target is defined first, and a solution then identified computationally. Essentially, this is also a global-optimisation problem, but where the target is not the minimum-energy crystal structure, but the structure and composition that provide the target property or collection of descriptors that define the figure of merit. Multi-objective optimisation procedures have been developed for this purpose.^31^ In principle, the chemical composition and crystal structure could both be varied in this search. The optimisation of the external (nuclear) potentials that give rise to a target property has been applied to molecular design in the form of linear combinations of atomic potentials.^32^ Such an ‘alchemical’ approach can be used to provide a continuous property surface, which can provide new insights into the relationship between properties and composition.

Due to the complexity of the inverse-design problem, it represents fertile ground for advanced machine-learning procedures, where the algorithm can adapt to the nature of the chemical systems being explored. Techniques such as artificial neural networks and representation learning have the potential to significantly enhance the discovery of functional materials. While this domain is largely in development, there have been several recent successful reports of tightly-integrated theory, computation and combinatorial experiments along these lines, including the first report of the 18-electron compound TaCoSn in a zinc-blende derived crystal structure,^33^ and the high-performance p-type transparent conductor Li-doped Cr_2_MnO_4_.^34^

## 3. The design process

A typical development process incorporates four stages, from setting the requirements to the design, development and testing of the product. In this section, we explore each step in the context of computational materials design.

### Requirements

The fundamental properties required are dictated by the specific application. These are discussed below in the development of materials for thermoelectric and photovoltaic devices, which represent two active contemporary research topics in materials chemistry and physics. For major applications, specific targets are commonly set by government or funding agencies (*e.g.* gravimetric and volumetric capacities for hydrogen storage), although these are not necessarily realistic and are subject to variation.

In most cases where multiple criteria have to be satisfied, a hierarchy of needs must be set. This could be in the form of a figure of merit, built up from a combination of weighted descriptors that favour low-level needs such as thermodynamic stability over high-level requirements such as cost and complexity. For heat-to-electricity conversion in thermoelectric devices, there is a well-established figure of merit (*ZT*) with calculable components, while for solar energy conversion it becomes difficult to construct an all-encompassing metric based on the properties of the bulk materials alone (see Section 5).

### Design

The challenge in design is addressing how to formulate a material, as defined by a chemical composition and crystal structure, to meet the set requirements. The almost infinite number of possibilities must be narrowed down to a tractable set using the tools previously described.

The introduction of constraints is useful for reducing the physical search space. These could be imposed by limiting the search to a smaller number of elements based on cost, availability and toxicity, as required for the intended application. It is also possible to limit the structural space, *e.g.* to combinations of metal oxide octahedra and tetrahedra as the structural building blocks. Efficient searching of the available phase space could be facilitated by means of a combinatorial optimisation algorithm such as the set of branch-and-bound methods.

There is an important distinction between screening and design. The former concerns searching for a solution over a large phase space, while the latter implies the use of existing knowledge or forward thinking. An effective materials-design procedure should employ known chemical principles – in magnetism for example, the connectivity required to promote electron-exchange interactions is well understood, while in ionic solids the electronegativity of the components is key to determining stability and chemical hardness.

It is difficult to avoid the influence of existing archetypes, *e.g.* for the photoelectrochemical splitting of water, TiO_2_ and its derivatives such as SrTiO_3_ have been intensively studied, yielding four decades of information on materials performance and limitations. From these studies, it is known that a d^0^ cation can be beneficial for reduction processes, owing to the long lifetime of the photoexcited electrons, which is required due to the slow kinetics of electron-transfer reactions.^35^ Even with a highly-optimised screening algorithm, a better set of inputs will more efficiently and more reliably provide a better set of solutions. The application of design to a constrained physical search space is explored in the section on solar cells.

### Development

At the development stage, the ideas originating from the design procedure can be translated into actual materials. Ultimately, there must be a cost-benefit analysis in terms of the computations. Often several rounds of screening based on the established hierarchy of requirements is a beneficial approach, *i.e.* if a material is calculated to be highly unstable thermodynamically, there is no need to do further calculations to establish its properties. If the calculation of a certain property requires thousands of processing hours, and it cannot be implemented at the design stage, then it could be used for secondary screening of candidates that emerge from the design procedure. The secondary screening would then employ a set of more rigorous simulation techniques, with the aim of producing a reliable set of final candidate materials. Such an approach is discussed in the context of thermoelectrics in Section 4.

The candidate materials that make it to the development stage should themselves be carefully analysed in terms of chemical bonding and crystal structure. If similar characteristics evolve independently across multiple systems, this may signify convergence towards a transferable design principle.

To aid comparison between different studies, reliable benchmarks need to be available. With the exception of simple properties such as lattice constants and cohesive energies, there is thus far a lack of consistency and open data in the field of computational materials science. In order to reproduce a result, the crystal structure and program input should be provided, and both rarely are. There are a number of fragmented computational property databases for specific applications or properties, *e.g.* thermoelectrics (; http://www.aflowlib.org), renewable energy (; http://materials.nrel.gov) and phonons (; http://phonondb.mtl.kyoto-u.ac.jp), while several specialist data repository infrastructures are starting to appear (; http://nomad-lab.eu, http://oqmd.org, ; http://www.aiida.net).

### Testing

To ensure that the candidate materials meet the design requirements, it is essential to test and validate as many characteristics as possible. A feedback loop may be required with experiment, which, rather than simply iterate the design process, also modifies it to maximise the overlap between theory and measurement. This stage could include providing spectral signatures (*e.g.* IR and Raman peak positions and intensities), insights into the finite-temperature behaviour using molecular dynamics simulations, and data on the preferred crystal terminations and the effect of morphology on the physical properties.

## 4. Thermoelectrics

The thermoelectric effect is the direct conversion of a temperature difference to an electrical voltage, and *vice versa*. Efficient thermoelectric materials would therefore make it possible to recover the large amounts of energy that are currently lost as heat during energy production and in industrial processes. This has fuelled the field of thermoelectrics and made it a fertile ground for materials prediction, not least because the figure of merit, *ZT*, is amenable to calculation. The ability of a material to convert heat to electricity can be formulated as a dimensionless quantity:
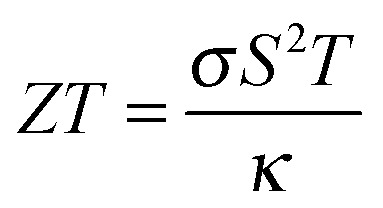
The Seebeck coefficient, *S*, and electrical conductivity, *σ*, which together determine the power factor, *S* ^2^ *σ*, can be approximated from the calculated electronic density of states under the assumption of a certain free carrier concentration (Fermi level). The denominator is the thermal conductivity, which should be minimised in an effective thermoelectric material. A pragmatic procedure combining semi-empirical and first-principles calculations to construct *ZT* has been applied to several hundred compounds, which can reproduce the salient features of the experimental literature.^36^

While computing the electronic components is now routine and easy to screen against, the vibrational component represents a greater challenge for simulations. To model the lattice thermal conductivity of a material, one must consider its lattice dynamics. The typical approach is to consider harmonic phonons; however, to model thermal conductivity the calculations must also account for the anharmonic effects (phonon–phonon interactions) that lead to finite phonon lifetimes.^37^ Due to the computational expense of computing many-phonon processes directly, it has become commonplace to employ simpler phenomenological models. However, as explored for the lead chalcogenides,^38^ first-principles methods can provide an accurate description of anharmonic lattice dynamics at a manageable computational cost. In addition to modelling phonon spectra and lattice thermal conductivity, these calculations can also predict quantitatively the temperature dependence of material structure and properties, which can be an important consideration for high-temperature applications such as thermoelectric generators.

*Ab initio* lattice-dynamics calculations can be expensive for large, low-symmetry unit cells, but are usually sufficiently tractable to be incorporated into late-stage ranking of candidate materials alongside, for example, accurate electronic-structure calculations. The absence of negative-frequency (imaginary) phonon modes can also be used to confirm the dynamical (as opposed to energetic) stability of a material.

The link between composition, structure and lattice thermal conductivity is not presently well understood, and no established engineering strategies exist for minimising it. Current benchmark thermoelectrics tend to fall into one of three categories: (i) materials composed of heavy atoms (*e.g.* PbTe), which exhibit naturally soft lattice vibrations (phonons) that promote strong phonon–phonon scattering at finite temperature; (ii) materials with strongly anharmonic lattice dynamics (*e.g.* some perovskite-structured materials with displacive symmetry-breaking instabilities); and (iii) materials modified by doping, alloying or nano-structuring. However, it is not clear which of these observations translate into general design principles. A case in point is the recent demonstration that SnSe, a compound with a relatively simple structure and stiffer chemical bonds than PbTe, has a considerably lower lattice thermal conductivity.^39^

**Fig. 3:**
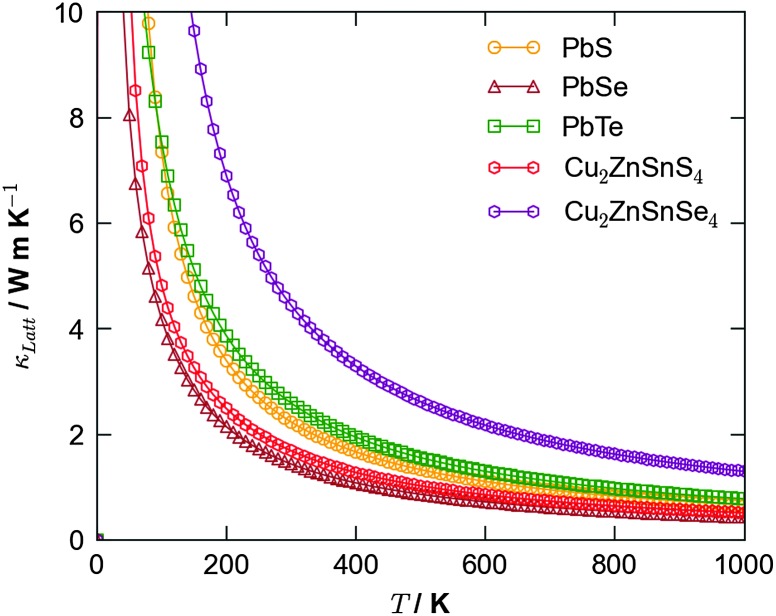
Calculated lattice thermal conductivity using anharmonic lattice dynamics as a function of temperature for the binary lead chalcogenides, PbS, PbSe and PbTe,^38^ and the quaternary semiconductors Cu_2_ZnSnS_4_ and Cu_2_ZnSnSe_4_.^40^

An alternative potential strategy is to investigate multicomponent systems in which the variation in atomic mass and bond strength should naturally dampen thermal conductivity. Since an ideal thermoelectric material should also be a good semiconductor, a potential route for exploration is the ternary and quaternary alloys that are currently being trialled as earth-abundant materials for photovoltaics, *e.g.* kesterite (Cu_2_ZnSnS_4_; CZTS) and the selenide analogue (Cu_2_ZnSnSe_4_; CZTSe). Recent simulations^40^ confirmed CZTS to possess a low lattice thermal conductivity competitive with that of PbTe, suggesting this to be a good route for future study (Fig. 3).

## 5. Thin-film solar cells

Heat-to-electricity conversion is useful for recovering waste energy. For primary energy production, the direct conversion of solar energy to electricity represents a sustainable and scalable approach. The solar resource is vastly larger than any other renewable-energy source. Building upon the success of silicon and second-generation thin-film photovoltaics (*i.e.* CdTe and CuInSe_2_), there is intense interest in the development of new photovoltaic materials. The target properties for an economic technology are earth abundance and non-toxicity of the constituent elements, and long lifetime of the full system.^41^ These are in addition to the basic requirements for photovoltaic action: a suitable optical band gap, and a balance between optical absorption, long charge-carrier lifetimes and moderate charge-carrier mobility.

The Shockley–Queisser limit directly links the band gap of a semiconductor to its maximum light-to-electricity conversion efficiency, under the assumptions of full light absorption, loss of excess photon energy and no other losses. This limit amounts to ∼33% efficiency for a single-junction solar cell with the standard AM1.5 solar spectrum. In a thin film (<5 μm), interference effects lead to a frequency-dependent optical absorption coefficient. Yu and Zunger^42^ formulated a simple metric based on this absorption-limited efficiency, and applied it to the screening of ternary Cu based photovoltaic absorber layers; a similar procedure was also reported by Oba and co-workers and applied to ZnSnP_2_ and CdSnP_2_.^43^

In practice, a photovoltaic device is more than just the bulk photoactive material. Critical factors include bulk defects, surface defects, morphology, interface reactions, and the electrical contacting. Device optimisation is considerably more difficult than the identification of an active material. Indeed, despite decades of research effort, there are numerous examples of materials with ideal bulk properties, but poor conversion efficiencies, *e.g.* Cu_2_O, SnS and FeS_2_.

In 2009, we explored a large family of tetrahedral semiconductors inspired by the work of Pamplin^28^ on multi-component materials. We systematically investigated charge-conserving cross substitutions of cations along the transition from binary, to ternary, to quaternary semiconductors.^44^ All structures considered were superlattices of the zincblende archetype. The ground-state configurations found for each of the quaternary I_2_–II–VI–VI_4_ materials are based on the kesterite and stannite mineral structures. The materials Cu_2_ZnSnS_4_ and Cu_2_ZnSnSe_4_ were subsequently widely studied for thin-film solar cells, with a current champion efficiency of 12.6%.^45^

The discovery of high-efficiency solution-processed solar cells based on hybrid halide perovskites has changed the face of contemporary photovoltaic research.^46^ Materials such as methylammonium lead iodide (CH_3_NH_3_PbI_3_) have the ability to efficiently separate photogenerated electrons and holes, seemingly independently of the device architecture or the material quality. In addition to their optimal physical characteristics (optical absorption and electrical conductivity), these materials display high dielectric permittivity,^47^ ferroelectric behaviour,^48^ and ionic conductivity.^49^ Despite over 2000 publications in the field over a short period of time, there is thus far no widely-accepted explanation as to why these materials are so effective for light-to-electricity conversion.

Beyond the most widely-studied methylammonium and formamidinium systems, there is a large family of hybrid organic–inorganic perovskites^50,51^ which will provide a fertile ground for materials discovery. Already, Jacobsen and co-workers have screened 240 inorganic and hybrid perovskites with a range of cations and anions, and found that the band gaps obey the expected chemical trends,^52^ *e.g.* the valence band energy can be controlled by the change in anion from the low binding-energy 5p orbitals of iodine to the high binding-energy 3p orbitals of Cl. Lessons learned from the success of methylammonium lead iodide have also been applied to the discovery of new systems. For example, by partially replacing the halide ions by thiocyanate, the thermodynamic stability has been predicted to be enhanced.^53^ One perspective discussed exploitation of lattice polarisation in solar cells (in so-called photoferroics),^54^ while another focused on the concept of defect tolerance, which can be aided by a large static dielectric constant to provide effective screening for photo-generated electrons and holes.^55^ The requirement of a divalent cation for halide perovskites can also be relaxed by forming so-called double perovskite structures, where Pb(ii) is replaced by an equivalent number of monovalent and trivalent cations (*e.g.* Ag and Bi). These suggestions are likely to result in large-scale explorations of novel photoactive materials systems in the near term.

As discussed above, a significant barrier in the design of novel photovoltaic systems is in the translation from materials to devices. The abundance of candidate materials and the paucity of efficient devices emphasises the importance of integrating bulk and extended defects, including surfaces and interfaces, into the later stages of the design procedure. Such information could be used to parameterise realistic device models as part of a multi-scale photovoltaic design procedure.

## 6. Conclusions

First-principles materials design is a research field still in its infancy. The tools required to make robust predictions are currently being assembled, with the potential to address issues of global significance. It is not simply a problem of computer science: input from materials chemists and physicists is required to develop appropriate application descriptors, to articulate clear design principles, and to interpret the results. Ultimately, an expert system that could autonomously identify functional materials on demand is realistic if the community can unite in the development of a common knowledge base and a powerful inference engine.

Our discussion was largely based on the properties of ordered bulk crystals. The treatment of disorder, including glassy materials, is still challenging for first-principles modelling, as is the description of extended defects such as grain boundaries and dislocations. The limitations of the models and simulations need to be considered when comparing to experimental results, and when choosing or eliminating candidate compounds.

In his seminal work on crystal-structure determination,^1^ Pauling emphasised the rule of parsimony, *i.e.* that things usually behave in the simplest or most economical manner. In materials design, simplicity is preferred to complexity, and the materials of interest should be feasible to calculate, synthesise, and characterise. A fanciful material consisting of a dozen components in unstable oxidation states may attract fundamental interest in its properties, but will have no lasting impact. In most cases it is a satisfactory solution, not the optimal solution that is required.
