# Supplementary material for: Computational materials design of crystalline solids
Source: Chem Soc Rev. 2016 Mar 18;45(22):6138–46. doi: 10.1039/c5cs00841g (PMC5103860; doi:10.1039/c5cs00841g)
Supplement: Supplementary file 1 [file CS-045-C5CS00841G-s001.pdf]

Electronic Supplementary Material (ESI) for Chemical Society Reviews.
This journal is © The Royal Society of Chemistry 2016

Computational Materials Design of Crystalline Solids

Keith T. Butler[1], Jarvist M. Frost[1], Jonathan M. Skelton[1], Katrine L. Svane[1], Aron Walsh[1,2]*

[1]Centre for Sustainable Chemical Technologies and Department of Chemistry, University of Bath, UK

[2] Global E^3 Institute and Department of Materials Science and Engineering, Yonsei University, Seoul, Korea

Extended Reading List

A snapshot of the Mendeley Group on Materials Design: https://www.mendeley.com/groups/8113991/materials-design

(1) Pauling, L. The Principles Determining the Structure of Complex Ionic Crystals. *J. Am. Chem. Soc.* **1929**, *51*, 1010–1026.

(2) Goldschmidt, V. M. The Principles of Distribution of Chemical Elements in Minerals and Rocks. *J. Chem. Soc.* **1937**, 655.

(3) Goodman, C. H. L. The Prediction of Semiconducting Properties in Inorganic Compounds. *J. Phys. Chem. Solids* **1958**, *6* (4), 305–314.

(4) Shockley, W.; Queisser, H. J. Detailed Balance Limit of Efficiency of P-N Junction Solar Cells. *J. Appl. Phys.* **1961**, *32* (3), 510.

(5) Pamplin, B. R. A Systematic Method of Deriving New Semiconducting Compounds by Structural Analogy. *J. Phys. Chem. Solids* **1964**, *25* (7), 675–684.

(6) Phillips, J. *Bonds and Bands in Semiconductors*; Academic Press: New York, 1973.

(7) Shannon, R. D. Revised Effective Ionic Radii and Systematic Studies of Interatomic Distances in Halides and Chalcogenides. *Acta Crystallogr. Sect. A* **1976**, *32* (5), 751–767.

(8) Thévet, F.; Dagron, C.; Flahaut, J. Contributionàl'étude Du Système Formépar L'étain, Le Soufre et L'iode. Mise Enévidence Des Deux Variétés de L'iodosulfure Stanneux Sn_2SI_2: Comportement Thermique Etétude Structurale. *J. Solid State Chem.* **1976**, *18* (2), 175–182.

(9) Bach, H.; Küppers, H. Cadmium Diiodate. *Acta Crystallogr. Sect. B* **1978**, *34* (1), 263–265.

(10) Butler, M. A.; Ginley, D. S. Prediction of Flatband Potentials at Semiconductor-Electrolyte Interfaces from Atomic Electronegativities. *J. Electrochem. Soc.* **1978**, *125* (2), 228–232.

(11) Chelikowsky, J. R.; Phillips, J. C. Quantum-Defect Theory of Heats of Formation and Structural Transition Energies of Liquid and Solid Simple Metal Alloys and Compounds. *Phys. Rev. B* **1978**, *17* (6), 2453–2477.

(12) Zunger, A. Systematization of the Stable Crystal Structure of All AB -Type Binary Compounds: A Pseudopotential Orbital-Radii Approach. *Phys. Rev. B* **1980**, *22* (12), 5839–5872.

(13) Pettifor, D. G. The Structures of Binary Compounds. I. Phenomenological Structure Maps. *J. Phys. C Solid State Phys.* **1986**, *19* (3), 285–313.

(14) Wimmer, E. Computational Materials Design: A Perspective for Atomistic Approaches. *J. Comput. Mater. Des.* **1994**, *1* (1993), 215–242.

(15) Harding, J. H.; Pyper, N. C. The Meaning of the Oxygen Second-Electron Affinity and Oxide Potential Models. *Philos. Mag. Lett.* **1995**, *71* (2), 113–121.

(16) Baumeister, W.; Vogell, W.; Meyers, B. L.; Decher, G.; Stuart, M. A. C.; Fleer, G.; Aust, E.; Meyer, W.; Knoll, W.; Schlenoff, J. B.; et al. Computational Design of Hierarchically Structured Materials. *Science* **1997**, *277* (August), 5330.

(17) Rao, C. N. R.; Gopalakrishnan, J. *New Directions in Solid State Chemistry*; Cambridge University Press, 1997.

(18) Kiselyova, N. N.; Gladun, V. P.; Vashchenko, N. D. Computational Materials Design Using Artificial Intelligence Methods. *J. Alloys Compd.* **1998**, *279*, 8–13.

(19) Villars, P.; Onodera, N.; Iwata, S. The Linus Pauling File (LPF) and Its Application to Materials Design. *J. Alloys Compd.* **1998**, *279* (1), 1–7.

(20) Ashby, M. F. Multi-Objective Optimization in Material Design and Selection. *Acta Mater.* **2000**, *48* (1), 359–369.

(21) Kiselyova, N. N. Databases and Semantic Networks for the Inorganic Materials Computer Design. *Eng. Appl. Artif. Intell.* **2000**, *13* (5), 533–542.

(22) Mitzi, D. B. Templating and Structural Engineering in Organic-Inorganic Perovskites. *J. Chem. Soc. Dalt. Trans.* **2001**, *2001* (1), 1–12.

(23) Simpson, T. W.; Mauery, T. M.; Korte, J.; Mistree, F. Kriging Models for Global Approximation in Simulation-Based Multidisciplinary Design Optimization. *AIAA J.* **2001**, *39* (12), 2233–2241.

(24) Mellot-Draznieks, C.; Girard, S.; Férey, G.; Schön, J. C.; Cancarevic, Z.; Jansen, M. Computational Design and Prediction of Interesting Not-yet-Synthesized Structures of Inorganic Materials by Using Building Unit Concepts. *Chem. - A Eur. J.* **2002**, *8* (18), 4102–4113.

(25) Sato, K.; Katayama-Yoshida, H. First Principles Materials Design for Semiconductor Spintronics. *Semicond. Sci. Technol.* **2002**, *17* (4), 367–376.

(26) Schaak, R. E.; Mallouk, T. E. Perovskites by Design: A Toolbox of Solid-State Reactions. *Chem. Mater.* **2002**, *14* (4), 1455–1471.

(27) Clarke, D. R.; Levi, C. G. Materials Design for the Next Generation Thermal Barrier Coatings. *Annu. Rev. Mater. Res.* **2003**, *33* (1), 383–417.

(28) Van de Walle, C. G.; Neugebauer, J. Universal Alignment of Hydrogen Levels in Semiconductors, Insulators and Solutions. *Nature* **2003**, *423* (6940), 626–628.

(29) Matsushita, H.; Katsui, A. Materials Design for Cu-Based Quaternary Compounds Derived from Chalcopyrite-Rule. *J. Phys. Chem. Solids* **2005**, *66* (11), 1933–1936.

(30) Hafner, J.; Wolverton, C.; Ceder, G. Toward Computational Materials Design: The Impact of Density Functional Theory on Materials Research. *MRS Bull.* **2006**, *31* (September), 659–668.

(31) Wang, M.; Hu, X.; Beratan, D. N.; Yang, W. Designing Molecules by Optimizing Potentials. *J. Am. Chem. Soc.* **2006**, *128* (10), 3228–3232.

(32) Woodley, S. M.; Catlow, R. Crystal Structure Prediction from First Principles. *Nat. Mater.* **2008**, *7* (12), 937–946.

(33) Chen, S.; Gong, X. G.; Walsh, A.; Wei, S.-H. Electronic Structure and Stability of Quaternary Chalcogenide Semiconductors Derived from Cation Cross-Substitution of II-VI and I-III-VI2 Compounds. *Phys. Rev. B* **2009**, *79*, 165211.

(34) Kudo, A.; Miseki, Y. Heterogeneous Photocatalyst Materials for Water Splitting. *Chem. Soc. Rev.* **2009**, *38* (1), 253–278.

(35) Kuehmann, C. J.; Olson, G. B. Computational Materials Design and Engineering. *Mater. Sci. Technol.* **2009**, *25* (4), 472–478.

(36) Oganov, A. R.; Chen, J.; Gatti, C.; Ma, Y.; Ma, Y.; Glass, C. W.; Liu, Z.; Yu, T.; Kurakevych, O. O.; Vladimir, L. Ionic High-Pressure Form of Elemental Boron. *Nature* **2009**, *457* (7231), 1–13.

(37) Rajan, K.; Suh, C.; Mendez, P. F. Principal Component Analysis and Dimensional Analysis as Materials Informatics Tools to Reduce Dimensionality in Materials Science and Engineering. *Stat. Anal. Data Min.* **2009**, *1* (6), 361–371.

(38) Chen, W.-T.; Kuang, H.-M.; Chen, H.-L. Solid-State Syntheses, Crystal Structures and Properties of Two Novel Metal Sulfur chlorides—$Zn_6S_5Cl_2$ and $Hg_3ZnS_2Cl_4$. *J. Solid State Chem.* **2010**, *183* (10), 2411–2415.

(39) Chua, A. L.-S.; Benedek, N. a; Chen, L.; Finnis, M. W.; Sutton, A. P. A Genetic Algorithm for Predicting the Structures of Interfaces in Multicomponent Systems. *Nat. Mater.* **2010**, *9* (5), 418–422.

(40) Hautier, G.; Fischer, C. C.; Jain, A.; Mueller, T.; Ceder, G. Finding Natures Missing Ternary Oxide Compounds Using Machine Learning and Density Functional Theory. *Chem. Mater.* **2010**, *22* (12), 3762–3767.

(41) Setyawan, W.; Curtarolo, S. High-Throughput Electronic Band Structure Calculations: Challenges and Tools. *Comput. Mater. Sci.* **2010**, *49* (2), 299–312.

(42) Stoffel, R. P.; Wessel, C.; Lumey, M.-W.; Dronskowski, R. Ab Initio Thermocheistry of Solid-State Materials. *Angew. Chemie* **2010**, *49* (31), 5242–5266.

(43) Hautier, G.; Jain, A.; Ong, S. P.; Kang, B.; Moore, C.; Doe, R.; Ceder, G. Phosphates as Lithium-Ion Battery Cathodes: An Evaluation Based on High-Throughput Ab Initio Calculations. *Chem. Mater.* **2011**, *23* (0), 3495–3508.

(44) Lonie, D.; Zurek, E. XtalOpt: An Open – Source Evolutionary Algorithm for Crystal Structure Prediction. *Comput. Phys. Commun.* **2011**, *182* (2), 372–387.

(45) Oganov, A. R.; Lyakhov, A. O.; Valle, M. How Evolutionary Crystal Structure Prediction Works - and Why. *Acc. Chem. Res.* **2011**, *44* (3), 227–237.

(46) Pelatt, B. D.; Ravichandran, R.; Wager, J. F.; Keszler, D. A. Atomic Solid State Energy Scale. *J. Am. Chem. Soc.* **2011**, *133* (42), 16852–16860.

(47) Pickard, C. J.; Needs, R. J. Ab Initio Random Structure Searching. *J. Phys. Condens. Matter* **2011**, *23* (5), 053201.

(48) Trimarchi, G.; Peng, H.; Im, J.; Freeman, A. J.; Cloet, V.; Raw, A.; Poeppelmeier, K. R.; Biswas, K.; Lany, S.; Zunger, A. Using Design Principles to Systematically Plan the Synthesis of Hole-Conducting Transparent Oxides: Cu_3VO_4 and Ag_3VO_4 as a Case Study. *Phys. Rev. B - Condens. Matter Mater. Phys.* **2011**, *84* (16), 1–14.

(49) Castelli, I. E.; Landis, D. D.; Thygesen, K. S.; Dahl, S.; Chorkendorff, I.; Jaramillo, T. F.; Jacobsen, K. W. New Cubic Perovskites for One- and Two-Photon Water Splitting Using the Computational Materials Repository. *Energy Environ. Sci.* **2012**, *5*, 9034–9043.

(50) Chen, H.; Hautier, G.; Jain, A.; Moore, C. Carbonophosphates: A New Family of Cathode Materials for Li-Ion Batteries Identified Computationally. *Chem. Mater.* **2012**, *24* (11), 2009–2016.

(51) Chen, S.; Wang, L.-W. Thermodynamic Oxidation and Reduction Potentials of Photocatalytic Semiconductors in Aqueous Solution. *Chem. Mater.* **2012**, *24* (18), 3659–3666.

(52) Curtarolo, S.; Setyawan, W.; Hart, G. L. W.; Jahnatek, M.; Chepulskii, R. V.; Taylor, R. H.; Wang, S.; Xue, J.; Yang, K.; Levy, O.; et al. AFLOW: An Automatic Framework for High-Throughput Materials Discovery. *Comput. Mater. Sci.* **2012**, *58*, 218–226.

(53) Graetzel, M.; Janssen, R. A. J.; Mitzi, D. B.; Sargent, E. H. Materials Interface Engineering for Solution-Processed Photovoltaics. *Nature* **2012**, *488* (7411), 304–312.

(54) Hautier, G.; Jain, A.; Ong, S. P. From the Computer to the Laboratory: Materials Discovery and Design Using First-Principles Calculations. *J. Mater. Sci.* **2012**, *47* (21), 7317–7340.

(55) Lee, M. M.; Teuscher, J.; Miyasaka, T.; Murakami, T. N.; Snaith, H. J. Efficient Hybrid Solar Cells Based on Meso-Superstructured Organometal Halide Perovskites. *Science* **2012**, *338* (6107), 643–647.

(56) Wang, Y.; Lv, J.; Zhu, L.; Ma, Y. CALYPSO: A Method for Crystal Structure Prediction. *Comput. Phys. Commun.* **2012**, *183* (10), 2063–2070.

(57) Yu, L.; Kokenyesi, R. S.; Keszler, D. A.; Zunger, A. Inverse Design of High Absorption Thin-Film Photovoltaic Materials. *Adv. Energy Mater.* **2012**, *3* (1), 43–38.

(58) Yu, L.; Zunger, A. Identification of Potential Photovoltaic Absorbers Based on First-Principles Spectroscopic Screening of Materials. *Phys. Rev. Lett.* **2012**, *108* (6), 068701.

(59) Zhang, X.; Stevanović, V.; D'Avezac, M.; Lany, S.; Zunger, A. Prediction of A_2BX_4 Metal-Chalcogenide Compounds via First-Principles Thermodynamics. *Phys. Rev. B* **2012**, *86* (1), 014109.

(60) Zhang, X.; Yu, L.; Zakutayev, A.; Zunger, A. Sorting Stable versus Unstable Hypothetical Compounds: The Case of Multi-Functional ABX Half-Heusler Filled Tetrahedral Structures. *Adv. Funct. Mater.* **2012**, *22* (7), 1425–1435.

(61) Bérubé, N.; Gosselin, V.; Gaudreau, J.; Côté, M. Designing Polymers for Photovoltaic Applications Using Ab Initio Calculations. *J. Phys. Chem. C* **2013**, *117* (16), 7964–7972.

(62) Booth, G. H.; Grüneis, A.; Kresse, G.; Alavi, A. Towards an Exact Description of Electronic Wavefunctions in Real Solids. *Nature* **2013**, *493* (7432), 365–370.

(63) Brivio, F.; Walker, A. B.; Walsh, A. Structural and Electronic Properties of Hybrid Perovskites for High-Efficiency Thin-Film Photovoltaics from First-Principles. *APL Mater.* **2013**, *1* (4), 042111.

(64) Curtarolo, S.; Hart, G. L. W.; Nardelli, M. B.; Mingo, N.; Sanvito, S.; Levy, O. The High-Throughput Highway to Computational Materials Design. *Nat. Mater.* **2013**, *12* (3), 191–201.

(65) Hautier, G.; Miglio, A.; Ceder, G.; Rignanese, G.-M.; Gonze, X. Identification and Design Principles of Low Hole Effective Mass P-Type Transparent Conducting Oxides. *Nat. Commun.* **2013**, *4*, 2292.

(66) Jain, A.; Castelli, I. E.; Hautier, G.; Bailey, D. H.; Jacobsen, K. W. Performance of Genetic Algorithms in Search for Water Splitting Perovskites. *J. Mater. Sci.* **2013**, *48* (19), 6519–6534.

(67) Jain, A.; Ong, S. P.; Hautier, G.; Chen, W.; Richards, W. D.; Dacek, S.; Cholia, S.; Gunter, D.; Skinner, D.; Ceder, G.; et al. Commentary: The Materials Project: A Materials Genome Approach to Accelerating Materials Innovation. *APL Mater.* **2013**, *1* (1), 011002.

(68) Lejaeghere, K.; Cottenier, S.; Van Speybroeck, V. Ranking the Stars: A Refined Pareto Approach to Computational Materials Design. *Phys. Rev. Lett.* **2013**, *111* (7), 075501.

(69) Meredig, B.; Wolverton, C. A Hybrid Computational-Experimental Approach for Automated Crystal Structure Solution. *Nat. Mater.* **2013**, *12* (2), 123–127.

(70) Montavon, G.; Rupp, M.; Gobre, V.; Vazquez-Mayagoitia, A.; Hansen, K.; Tkatchenko, A.; Müller, K.-R.; Anatole von Lilienfeld, O. Machine Learning of Molecular Electronic Properties in Chemical Compound Space. *New J. Phys.* **2013**, *15* (9), 095003.

(71) Ong, S. P.; Richards, W. D.; Jain, A.; Hautier, G.; Kocher, M.; Cholia, S.; Gunter, D.; Chevrier, V. L.; Persson, K. A.; Ceder, G. Python Materials Genomics (Pymatgen): A Robust, Open-Source Python Library for Materials Analysis. *Comput. Mater. Sci.* **2013**, *68*, 314–319.

(72) Peng, H.; Zakutayev, A.; Lany, S.; Paudel, T. R.; D'Avezac, M.; Ndione, P. F.; Perkins, J. D.; Ginley, D. S.; Nagaraja, A. R.; Perry, N. H.; et al. Li-Doped Cr_2MnO_4: A New P-Type Transparent Conducting Oxide by Computational Materials Design. *Adv. Funct. Mater.* **2013**, *23* (42), 5267–5276.

(73) Pettifor, D. G. Structure Maps For. Pseudobinary and Ternary Phases. *Mater. Sci. Technol.* **1988**, *4* (8), 675–691.

(74) Pilania, G.; Wang, C.; Jiang, X.; Rajasekaran, S.; Ramprasad, R. Accelerating Materials Property Predictions Using Machine Learning. *Sci. Rep.* **2013**, *3*, 2810.

(75) Yang, J.; Yip, H.-L.; Jen, A. K.-Y. Rational Design of Advanced Thermoelectric Materials. *Adv. Energy Mater.* **2013**, No. 2, 549–565.

(76) Yokoyama, T.; Oba, F.; Seko, A.; Hayashi, H.; Nose, Y.; Tanaka, I. Theoretical Photovoltaic Conversion Efficiencies of $ZnSnP_2$, $CdSnP_2$, and $Zn_{1-x}Cd_xSnP_2$ Alloys. *Appl. Phys. Express* **2013**, *6* (6), 4–7.

(77) Zakutayev, A.; Zhang, X.; Nagaraja, A.; Yu, L.; Lany, S.; Mason, T. O.; Ginley, D. S.; Zunger, A. Theoretical Prediction and Experimental Realization of New Stable Inorganic Materials Using the Inverse Design Approach. *J. Am. Chem. Soc.* **2013**, *135* (27), 10048–10054.

(78) Zhang, W.; Oganov, A. R.; Goncharov, A. F.; Zhu, Q.; Boulfelfel, S. E.; Lyakhov, A. O.; Stavrou, E.; Somayazulu, M.; Prakapenka, V. B.; Konôpková, Z. Unexpected Stable Stoichiometries of Sodium Chlorides. *Science* **2013**, *342*, 1502–1505.

(79) Armiento, R.; Kozinsky, B.; Hautier, G.; Fornari, M.; Ceder, G. High-Throughput Screening of Perovskite Alloys for Piezoelectric Performance and Thermodynamic Stability. *Phys. Rev. B* **2014**, *89* (13), 134103.

(80) Bahers, T. Le; Rérat, M.; Sautet, P. Semiconductors Used in Photovoltaic and Photocatalytic Devices: Assessing Fundamental Properties from DFT. *J. Phys. Chem. C* **2014**, *118* (12), 5997–6008.

(81) Bjerg, L.; Iversen, B. B.; Madsen, G. K. H. Modeling the Thermal Conductivities of the Zinc Antimonides ZnSb and Zn_4Sb_3. *Phys. Rev. B* **2014**, *89* (2), 024304.

(82) Butler, K. T.; Walsh, A. Ultra-Thin Oxide Films for Band Engineering: Design Principles and Numerical Experiments. *Thin Solid Films* **2014**, *559*, 64–68.

(83) Castelli, I. E.; Jacobsen, K. W. Designing Rules and Probabilistic Weighting for Fast Materials Discovery in the Perovskite Structure. *Model. Simul. Mater. Sci. Eng.* **2014**, *22* (5), 055007.

(84) Castelli, I. E.; García-Lastra, J. M.; Thygesen, K. S.; Jacobsen, K. W. Bandgap Calculations and Trends of Organometal Halide Perovskites. *APL Mater.* **2014**, *2* (8), 081514.

(85) Deacon-Smith, D. E. E.; Scanlon, D. O.; Catlow, C. R. A.; Sokol, A. A.; Woodley, S. M. Interlayer Cation Exchange Stabilizes Polar Perovskite Surfaces. *Adv. Mater.* **2014**, *26* (42), 7252–7256.

(86) Frost, J. M.; Butler, K. T.; Brivio, F.; Hendon, C. H.; Van Schilfgaarde, M.; Walsh, A. Atomistic Origins of High-Performance in Hybrid Halide Perovskite Solar Cells. *Nano Lett.* **2014**, *14* (5), 2584–2590.

(87) Frost, J. M.; Butler, K. T.; Walsh, A. Molecular Ferroelectric Contributions to Anomalous Hysteresis in Hybrid Perovskite Solar Cells. *APL Mater.* **2014**, *2* (8), 081506.

(88) Garrity, K. F.; Rabe, K. M.; Vanderbilt, D. Hyperferroelectrics: Proper Ferroelectrics with Persistent Polarization. *Phys. Rev. Lett.* **2014**, *112* (12), 127601.

(89) Jackson, A. J.; Walsh, A. Ab Initio Thermodynamic Model of Cu_2ZnSnS_4. *J. Mater. Chem. A* **2014**, *2*, 7829–7836.

(90) Meredig, B.; Agrawal, A.; Kirklin, S.; Saal, J. E.; Doak, J. W.; Thompson, A.; Zhang, K.; Choudhary, A.; Wolverton, C. Combinatorial Screening for New Materials in

Unconstrained Composition Space with Machine Learning. *Phys. Rev. B* **2014**, *89* (9), 094104.

(91) Shoemaker, D. P.; Hu, Y.-J.; Chung, D. Y.; Halder, G. J.; Chupas, P. J.; Soderholm, L.; Mitchell, J. F.; Kanatzidis, M. G. In Situ Studies of a Platform for Metastable Inorganic Crystal Growth and Materials Discovery. *Proc. Natl. Acad. Sci. U. S. A.* **2014**, *111* (30), 10922–10927.

(92) Skelton, J. M.; Parker, S. C.; Togo, A.; Tanaka, I.; Walsh, A. Thermal Physics of the Lead Chalcogenides PbS, PbSe, and PbTe from First Principles. *Phys. Rev. B* **2014**, *89*, 205203.

(93) Toher, C.; Plata, J. J.; Levy, O.; de Jong, M.; Asta, M.; Nardelli, M. B.; Curtarolo, S. High-Throughput Computational Screening of Thermal Conductivity, Debye Temperature, and Grüneisen Parameter Using a Quasiharmonic Debye Model. *Phys. Rev. B* **2014**, *90* (17), 174107.

(94) Urban, A.; Lee, J.; Ceder, G. The Configurational Space of Rocksalt-Type Oxides for High-Capacity Lithium Battery Electrodes. *Adv. Energy Mater.* **2014**, *4* (13), 1400478.

(95) Wang, W.; Winkler, M. T.; Gunawan, O.; Gokmen, T.; Todorov, T. K.; Zhu, Y.; Mitzi, D. B. Device Characteristics of CZTSSe Thin-Film Solar Cells with 12.6% Efficiency. *Adv. Energy Mater.* **2014**, *4* (7), 1301465.

(96) Zakutayev, A.; Allen, A. J.; Zhang, X.; Vidal, J.; Cui, Z.; Lany, S.; Yang, M.; DiSalvo, F. J.; Ginley, D. S. Experimental Synthesis and Properties of Metastable $CuNbN_2$ and Theoretical Extension to Other Ternary Copper Nitrides. *Chem. Mater.* **2014**, *26* (17), 4970–4977.

(97) Zhao, L.-D.; Lo, S.-H.; Zhang, Y.; Sun, H.; Tan, G.; Uher, C.; Wolverton, C.; Dravid, V. P.; Kanatzidis, M. G. Ultralow Thermal Conductivity and High Thermoelectric Figure of Merit in SnSe Crystals. *Nature* **2014**, *508* (7496), 373–377.

(98) Balachandran, P. V.; Theiler, J.; Rondinelli, J. M.; Lookman, T. Materials Prediction via Classification Learning. *Sci. Rep.* **2015**, *5*, 13285.

(99) Brandt, R. E.; Stevanović, V.; Ginley, D. S.; Buonassisi, T. Identifying Defect-Tolerant Semiconductors with High Minority Carrier Lifetimes: Beyond Hybrid Lead Halide Perovskites. *MRS Commun.* **2015**, *2* (5), 265–275.

(100) Buckeridge, J.; Butler, K. T.; Catlow, C. R. A.; Logsdail, A. J.; Scanlon, D. O.; Shevlin, S. A.; Woodley, S. M.; Sokol, A. A.; Walsh, A. Polymorph Engineering of TiO_2: Demonstrating How Absolute Reference Potentials Are Determined by Local Coordination. *Chem. Mater.* **2015**, *27* (11), 3844–3851.

(101) Butler, K. T.; Frost, J. M.; Walsh, A. Ferroelectric Materials for Solar Energy Conversion: Photoferroics Revisited. *Energy Environ. Sci.* **2015**, *8*, 838–848.

(102) Eames, C.; Frost, J. M.; Barnes, P. R. F.; O'Regan, B. C.; Walsh, A.; Islam, M. S. Ionic Transport in Hybrid Lead Iodide Perovskite Solar Cells. *Nat. Commun.* **2015**, *6*, 7497.

(103) Ganose, A. M.; Savory, C. N.; Scanlon, D. O. $(CH_3NH_3)_2Pb(SCN)_2I_2$: A New More Stable Structural Motif for Hybrid Halide Photovoltaics? *J. Phys. Chem. Lett.* **2015**, *6*, 4594–4598.

(104) Gautier, R.; Zhang, X.; Hu, L.; Yu, L.; Lin, Y.; Sunde, T. O. L.; Chon, D.; Poeppelmeier, K. R.; Zunger, A. Prediction and Accelerated Laboratory Discovery of Previously Unknown 18-Electron ABX Compounds. *Nat. Chem.* **2015**, *7*, 308–316.

(105) Isayev, O.; Fourches, D.; Muratov, E. N.; Oses, C.; Rasch, K.; Tropsha, A.; Curtarolo, S. Materials Cartography: Representing and Mining Material Space Using Structural and Electronic Fingerprints. *Chem. Mater.* **2015**, *27* (3), 735–743.

(106) Kieslich, G.; Sun, S.; Cheetham, T. An Extended Tolerance Factor Approach for Organic-Inorganic Perovskites. *Chem. Sci.* **2015**, *6*, 3430–3433.

(107) Li, Z.; Yang, M.; Park, J.-S.; Wei, S.-H.; Berry, J.; Zhu, K. Stabilizing Perovskite Structures by Tuning Tolerance Factor: Formation of Formamidinium and Cesium Lead Iodide Solid-State Alloys. *Chem. Mater.* **2015**, *28* (1), 284–292.

(108) Murray, A.; Frost, J. M.; Hendon, C.; Molloy, C. D.; Carbery, D.; Walsh, A. Modular Design of SPIRO-OMeTAD Analogues as Hole Transport Materials in Solar Cells. *Chem. Commun.* **2015**, *51*, 8935–8938.

(109) Rondinelli, J. M.; Poeppelmeier, K. R.; Zunger, A. Research Update: Towards Designed Functionalities in Oxide-Based Electronic Materials. *APL Mater.* **2015**, *3*, 080702.

(110) Skelton, J. M.; Jackson, A. J.; Dimitrievska, M.; Wallace, S. K.; Walsh, A. Vibrational Spectra and Lattice Thermal Conductivity of Kesterite-Structured Cu_2ZnSnS_4 and $Cu_2ZnSnSe_4$. *APL Mater.* **2015**, *3* (4), 041102.

(111) Togo, A.; Chaput, L.; Tanaka, I. Distribution of Phonon Lifetimes in Brillouin Zones. *Phys. Rev. B* **2015**, *91*, 094306.

(112) Walsh, A. The Quest for New Functionality. *Nat. Chem.* **2015**, *7* (4), 274–275.

(113) Wang, F.; Grinberg, I.; Jiang, L.; Young, S. M.; Davies, P. K.; Rappe, A. M. Materials Design of Visible-Light Ferroelectric Photovoltaics from First Principles. *Ferroelectrics* **2015**, *483* (1), 1–12.

(114) Yan, J.; Gorai, P.; Ortiz, B.; Miller, S.; Barnett, S. A.; Mason, T.; Stevanović, V.; Toberer, E. S. Material Descriptors for Predicting Thermoelectric Performance. *Energy Environ. Sci.* **2015**, *8*, 983–994.

(115) Yim, K.; Yong, Y.; Lee, J.; Lee, K.; Nahm, H.-H.; Yoo, J.; Lee, C.; Seong Hwang, C.; Han, S. Novel High-κ Dielectrics for Next-Generation Electronic Devices Screened by Automated Ab Initio Calculations. *NPG Asia Mater.* **2015**, *7* (6), e190.

(116) Liu, J.; Fernández-Serra, M. V.; Allen, P. B. Special Quasiordered Structures: Role of Short-Range Order in the Semiconductor Alloy $(GaN)_{1-x}(ZnO)_x$. *Phys. Rev. B* **2016**, *93* (5), 054207.

(117) Stevanovic, V.; Stevanović, V. Sampling Polymorphs of Ionic Solids Using Random Superlattices. *Phys. Rev. Lett.* **2016**, *116* (7), 075503.
